# Preoperative fluorescence in situ hybridization analysis as a predictor of tumor recurrence in patients with non-muscle invasive bladder cancer: a bi-institutional study

**DOI:** 10.1186/s12967-023-04528-2

**Published:** 2023-10-02

**Authors:** Junjiong Zheng, Sihong Lu, Yi Huang, Xu Chen, Jie Zhang, Yuhui Yao, Jinhua Cai, Jieying Wu, Jianqiu Kong, Tianxin Lin

**Affiliations:** 1 Department of Urology, Guangdong Provincial Key Laboratory of Malignant Tumor Epigenetics and Gene Regulation, Sun Yat-Sen Memorial Hospital, Sun Yat-Sen University, Guangdong Provincial Clinical Research Center for Urological Diseases, 107 Yan Jiang West Road, Guangzhou, People’s Republic of China; 2 Department of Neurology, Sun Yat-Sen Memorial Hospital, Sun Yat-Sen University, 107 Yan Jiang West Road, Guangzhou, People’s Republic of China; 3 Department of Urology, the Third Affiliated Hospital of Sun Yat-Sen University, No. 600 Tianhe Road, Guangzhou, People’s Republic of China

**Keywords:** Fluorescence in situ hybridization, Non-muscle invasive bladder cancer, Nomogram, Recurrence

## Abstract

**Background:**

Non-muscle invasive bladder cancer (NMIBC) is known for its elevated recurrence rate, necessitating an enhancement in the current risk stratification for recurrence. The urine-based fluorescence in situ hybridization (FISH) assay has emerged as a noninvasive auxiliary tool for detecting bladder cancer. The aim of this study was to explore the potential relationship between the preoperative FISH assay and recurrence, and to develop a FISH-clinical nomogram for predicting the recurrence-free survival (RFS) in NMIBC patients.

**Methods:**

In total, 332 eligible patients were enrolled from two hospitals. The SYSMH cohort was randomly assigned to the training set (n = 168) and the validation set I (n = 72) at a ratio of 7:3, while the SYSUTH cohort was allocated to the validation set II (n = 92). The correlation between the preoperative FISH assay and recurrence was determined through the Cox regression analysis. The least absolute shrinkage and selection operator (LASSO) Cox regression algorithm was used for model construction. The performance of the model was assessed by its discrimination, calibration, and clinical usefulness.

**Results:**

We uncovered that chromosome 7 aneuploidy, p16 locus loss, number of the positive FISH sites, and the FISH test result were significantly associated with tumor recurrence. Then, a FISH-clinical nomogram incorporating the FISH test result, T stage, associated CIS, tumor grade, and tumor status was developed. It showed favorable calibration and discrimination with a C-index of 0.683 (95%CI, 0.611–0.756) in the training set, which was confirmed in the validation set I and validation set II with C-indexes of 0.665 (95%CI, 0.565–0.765) and 0.778 (95%CI, 0.665–0.891), respectively. Decision curve analysis revealed the clinical usefulness of the nomogram. Moreover, our proposed nomogram significantly outperformed the guideline-recommended EORTC and CUETO scoring models.

**Conclusion:**

Our study confirmed the prognostic value of the preoperative FISH assay and proposed a FISH-clinical nomogram to predict RFS in NMIBC patients. Our nomogram can serve as a more precise tool for recurrence risk stratification, which may optimize disease management in bladder cancer and improve patient prognosis.

**Supplementary Information:**

The online version contains supplementary material available at 10.1186/s12967-023-04528-2.

## Background

Bladder cancer is one of the most common malignant tumors of the urinary system [1]. Approximately 75% of the newly diagnosed cases of bladder cancer are non-muscle invasive bladder cancer (NMIBC) [2]. NMIBC is a heterogeneous disease with widely different prognoses [3], and 50–70% of them will experience a recurrence following resection [4, 5]. NMIBC Therefore, it remains crucial to predict recurrence for NMIBC patients. The European Organisation for Research and Treatment of Cancer (EORTC) scoring model [6] and the Club Urologico Espanol de Tratamiento Oncologico (CUETO) scoring model [7] are recommended for evaluating the risk of recurrence for individual patients with NMIBC after transurethral resection of bladder tumor (TURBT) by the current European Association of Urology guidelines [3]. The scoring systems are attractively simple and are both based on several clinical and pathological factors. However, the prediction accuracy of the models remains to be further improved [8–10]. Thus, there is a great need to search for new and reliable predictors to improve the accuracy of prognostic prediction in NMIBC patients.

Fluorescence in situ hybridization (FISH) is used to detect genetic alterations most commonly associated with bladder cancer, which is a urine-based non-invasive test used in diagnosis as well as follow-up [11]. Previous studies have proved that complex detection of aneuploidy of chromosomes 3, 7, and 17 and a deletion of locus 9p21 could identify BC cells in exfoliated cells from voided or washing urine samples with high sensitivity and specificity [12, 13]. Notably, the FISH assay has higher sensitivity in the detection of bladder cancer than the conventional urine cytology test [14, 15]. Previously, our research has demonstrated that chromosome-specific centromeric probe 7 (CSP7) status measured by FISH assay is associated with muscular invasion in bladder cancer [16]. Meanwhile, previous studies have revealed that a positive FISH test after BCG intravesical perfusion correlated with a higher risk of recurrent tumor in patients with NMIBC at intermediate and high risk [17, 18]. Before undertaking this study, we searched PubMed using the terms “(((“bladder cancer”) OR (“bladder tumor”)) AND ((“fluorescence in situ hybridization”) OR (FISH))) AND (((prognosis) OR (“recurrence-free survival”)) OR (RFS))” to find research published in any language between Dec 1, 2000, and Dec 1, 2022. No studies on elucidating the association of the status of the FISH sites in the preoperative FISH test with tumor stage, grade, and recurrence in NMIBC patients were identified. Therefore, whether the preoperative FISH test can be used for the prediction of recurrence in patients with NMIBC is worthy of further investigation.

Hence, the present study is designed to figure out the correlation of preoperative FISH analysis with tumor grade, stage, and recurrence in patients with NMIBC. In addition, we attempted to incorporate the preoperative FISH test results and clinicopathological predictors to establish a nomogram for predicting the recurrence of NMIBC after surgery.

## Materials and methods

### Patients

Approval from the Institutional Review Boards was obtained for our retrospective study and the patient’s informed consent was waived. Overall, 240 patients treated at Sun Yat-Sen Memorial Hospital (SYSMH) between February 2015 and September 2020, and 92 patients treated at the Third Affiliated Hospital of Sun Yat-Sen University (SYSUTH) between June 2014 and September 2022 were enrolled in this study. The inclusion criteria in this study were (a) NMIBC confirmed by pathology; (b) underwent TURBT; (c) FISH test performed less than 14 days before surgery; (d) comprehensive clinicopathological data and follow-up information available. And the exclusion criteria were (a) concurrent presence of other cancer diseases; (b) diagnosis of non-urothelial carcinoma. The SYSMH cohort was randomly assigned to the training set (n = 168) and the validation set I (n = 72) at a ratio of 7:3, while the SYSUTH cohort was allocated to the validation set II (n = 92).

Baseline clinicopathological characteristics, including age, sex, tumor status (primary or recurrent tumor), tumor size, tumor number, and preoperative FISH assay results were obtained from the electronic medical record system. Pathology slides were independently reviewed by two experienced pathologists, and data derived from the slides were recorded, including tumor grade and pathologic T stage. Any disagreement was resolved by consultation. The Union for International Cancer Control 8th edition TNM staging system was used for pathologic tumor staging, while the WHO 2004/2016 grading system was used to classify the histologic grade of the tumor. Recurrence-free survival (RFS) was defined as the time from the date of surgery to the date of recurrence or the last follow-up.

### FISH assay

The FISH tests were conducted with commercially available kits (GP Medical Technologies, Ltd, Beijing, China) in both institutions. The detection procedure, including urine specimen collection, slides preparation, hybridization of fluorescent-labeled DNA probes, and signal detection, was described in considerable detail in Supplementary Methods [16]. In the FISH test, the status of the FISH site and the FISH result are reported based on specific criteria. When the frequency of aneuploidies on chromosomes 3, 7, and 17 or the frequency of p16 locus loss exceeds the diagnostic thresholds, the positivity of the FISH site is reported. And a positive result of the FISH test is given if two or more than two types of abnormal results with these probe signals are present, or only gene locus-specific probe p16 (GLP-p16) is positive.

### Exploration of the correlation of FISH assay with tumor grade, stage, and RFS

We used pie plots and bar plots to depict the distributions of the FISH assay results in the whole study cohort. Chi-square tests and logistic regression analyses were used to assess the correlations of FISH assay results with tumor grade and stage. In addition, Cox regression analyses were used to demonstrate the association between FISH assay results and RFS.

### Model construction and performance assessment

Least absolute shrinkage and selection operator (LASSO) algorithm is a powerful machine-learning method for variable selection [19]. In the training set, LASSO Cox regression analysis was utilized to select the most useful predictive variable from the FISH assay results and the candidate clinicopathological predictors. Then, a FISH-clinical nomogram was developed based on the regression analysis results. A risk score for each patient was calculated as a linear combination of the selected predictors weighted by their respective regression coefficients to reflect the risk of recurrence:

Risk score = a_1_P_1_ + a_2_P_2_ + … + a_i_P_i_, where P_i_ is the selected predictor, and a_i_ is the regression coefficient of P_i_.

The performance of the FISH-clinical nomogram was assessed with respect to its discrimination and calibration in the training set. To quantitatively evaluate the discriminative ability, Harrell’s C-index was calculated, and bootstrapping using 1000 resampling procedures was applied [20]. The calibration of the model was evaluated by conducting a consistency assessment between the actual recurrence probabilities and the predicted recurrence probabilities based on the prediction model.

### Validation of the nomogram

The validation set I and validation set II were used to validate the performance of the FISH-clinical nomogram. Risk scores were calculated for patients in the validation sets based on the regression equation obtained from the training set. Subsequently, the C-index and calibration curves were also applied to evaluate the performance of the nomogram. In addition, decision curve analysis (DCA) was conducted to estimate the clinical usefulness of the nomogram. Finally, our proposed FISH-clinical model was compared with the EORTC and CUETO scoring models.

### Categorization of patients into high or low risk groups

By using X-tile plots, an optimal risk score cutoff value was identified in the training set to divide all patients into high risk and low risk groups [21]. The difference in the survival curves of the high-risk and low-risk groups was assessed by using the log-rank test. Furthermore, stratified analyses were also performed according to the age and sex of the patients in the whole study cohort.

### Statistical analyses

X-tile plots were created by the X-tile software version 3.6.1 (Yale University School of Medicine, New Haven, CT, USA) [21]. With X-tile plots, the optimum cutoff can be automatically selected based on the highest χ² value (i.e., minimum *P* value) defined using Kaplan-Meier survival analysis and the log-rank test. Other statistical tests in our study were all conducted using R software version 4.0.4 (https://www.r-project.org/). R packages used in the study are described in Additional file 1: Table S1. All statistical tests were two-tailed, and *P* < 0.05 were deemed significant.

## Results

### Patient characteristics

The FISH assay results and patient clinicopathological characteristics of the training and validation sets are displayed in Table 1. In our study, the median follow-up time was 31.3 months (Interquartile range, 17.9–46.6). During the follow-up, there were 86 patients (25.9%) suffered from recurrence among the enrolled patients.

**Table 1:** Baseline clinicopathological characteristics of the patients enrolled in this study

	Whole study cohort (n = 332)	Training set (n = 168)	Validation set I (n = 72)	Validation set II (n = 92)
Age, years				
Median (IQR^†^)	63 (56, 72)	62 (54, 72)	62 (57, 71)	66 (56, 75)
Sex				
Male	283 (85.2%)	141 (83.9%)	63 (87.5%)	79 (85.9%)
Female	49 (14.8%)	27 (16.1%)	9 (12.5%)	13 (14.1%)
Tumor status				
Primary	290 (87.3%)	147 (87.5%)	57 (79.2%)	86 (93.5%)
Recurrent	42 (12.7%)	21 (12.5%)	15 (20.8%)	6 (6.5%)
Tumor size				
≤3 cm	250 (75.3%)	127 (75.6%)	51 (70.8%)	72 (78.3%)
>3 cm	82 (24.7%)	41 (24.4%)	21 (29.2%)	20 (21.7%)
Tumor number				
Single	202 (60.8%)	84 (50.0%)	38 (52.8%)	80 (87.0%)
Multiple	130 (39.2%)	84 (50.0%)	34 (47.2%)	12 (13.0%)
T stage				
Ta	194 (58.4%)	99 (58.9%)	39 (54.2%)	56 (60.9%)
T1	138 (41.6%)	69 (41.1%)	33 (45.8%)	36 (39.1%)
Associated CIS				
No	320 (96.4%)	160 (95.2%)	70 (97.2%)	90 (97.8%)
Yes	12 (3.6%)	8 (4.8%)	2 (2.8%)	2 (2.2%)
Grade				
Low	141 (42.5%)	63 (37.5%)	22 (30.6%)	56 (60.9%)
High	191 (57.5%)	105 (62.5%)	50 (69.4%)	36 (39.1%)
CSP3				
Negative	190 (57.2%)	103 (61.3%)	38 (52.8%)	49 (53.3%)
Positive	142 (42.8%)	65 (38.7%)	34 (47.2%)	43 (46.7%)
CSP7				
Negative	194 (58.4%)	105 (62.5%)	40 (55.6%)	49 (53.3%)
Positive	138 (41.6%)	63 (37.5%)	32 (44.4%)	43 (46.7%)
CSP17				
Negative	183 (55.1%)	95 (56.5%)	39 (54.2%)	49 (53.3%)
Positive	149 (44.9%)	73 (43.5%)	33 (45.8%)	43 (46.7%)
GLP-p16				
Negative	238 (71.7%)	134 (79.8%)	56 (77.8%)	48 (52.2%)
Positive	94 (28.3%)	34 (20.2%)	16 (22.2%)	44 (47.8%)
No. of positive FISH site				
0	135 (40.7%)	65 (38.7%)	27 (37.5%)	43 (46.7%)
1	53 (16.0%)	37 (22.0%)	11 (15.3%)	5 (5.4%)
2	15 (4.5%)	8 (4.8%)	5 (6.9%)	2 (2.2%)
3	76 (22.8%)	50 (29.7%)	22 (30.6%)	4 (4.4%)
4	53 (16.0%)	8 (4.8%)	7 (9.7%)	38 (41.3%)
FISH test				
Negative	156 (47.0%)	82 (48.8%)	31 (43.1%)	43 (46.7%)
Positive	176 (53.0%)	86 (51.2%)	41 (56.9%)	49 (53.3%)
Follow Time, months				
Median (IQR)	31.3 (17.9, 46.6)	34.8 (25.1, 47.6)	34.8 (21.9, 39.8)	23.8 (10.0, 44.1)
Recurrence				
No	246 (74.1%)	122 (72.6%)	48 (66.7%)	76 (82.6%)
Yes	86 (25.9%)	46 (27.4%)	24 (33.3%)	16 (17.4%)

Data are n or n (%) unless otherwise indicated

^†^*IQR* interquartile range

### Distributions of the FISH assay results

**Fig. 1:**
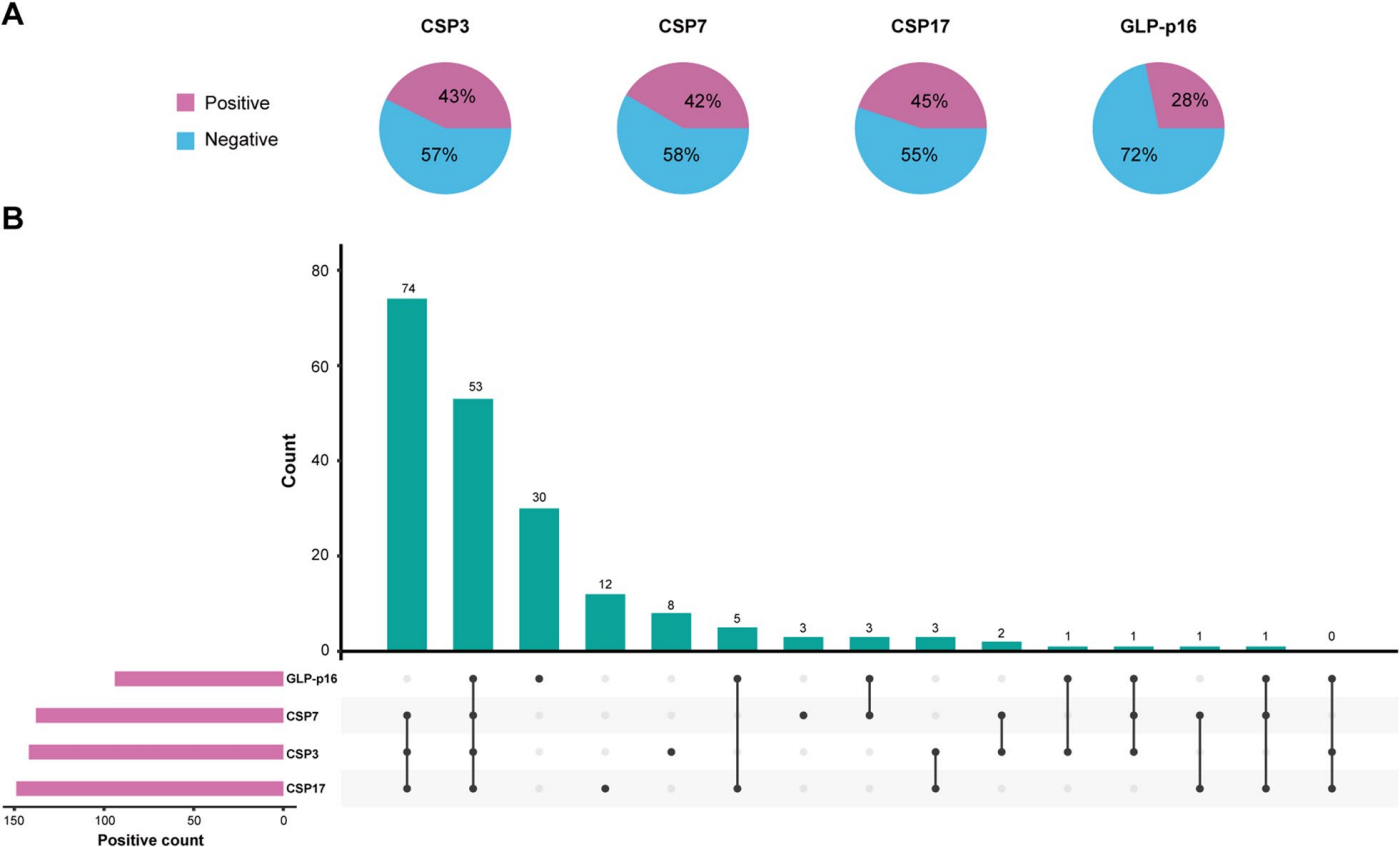
The distributions of the FISH assay result in all enrolled patients **A** Pie plots depicting the distributions of the status of four FISH sites. **B** Upset plot depicting the distributions of various types of positive FISH sites combination. The horizontal axis uses a connected dot plot to indicate different types of positive FISH sites combination, and the vertical axis presents the patient count for the specific combination. The horizontal red bars represent the positive count of each FISH site. *FISH* fluorescence in situ hybridization; *CSP* chromosome-specific centromeric probe; *GLP* gene locus-specific probe

The distributions of the status of the four FISH sites among all patients are shown in Fig. 1A. There were 43%, 42%, and 45% of patients had aneuploidy by chromosomes 3, 7, and 17, respectively, and 28% of patients had p16 locus loss. Meanwhile, as shown in Fig. 1B, the most common type of the FISH positive sites combination was CSP3 (+), CSP7 (+), CSP17 (+), and GLP-p16 (−), which was consistent with our previous study [16].

### Exploration of the correlation of FISH assay with tumor grade, stage, and RFS

**Fig. 2:**
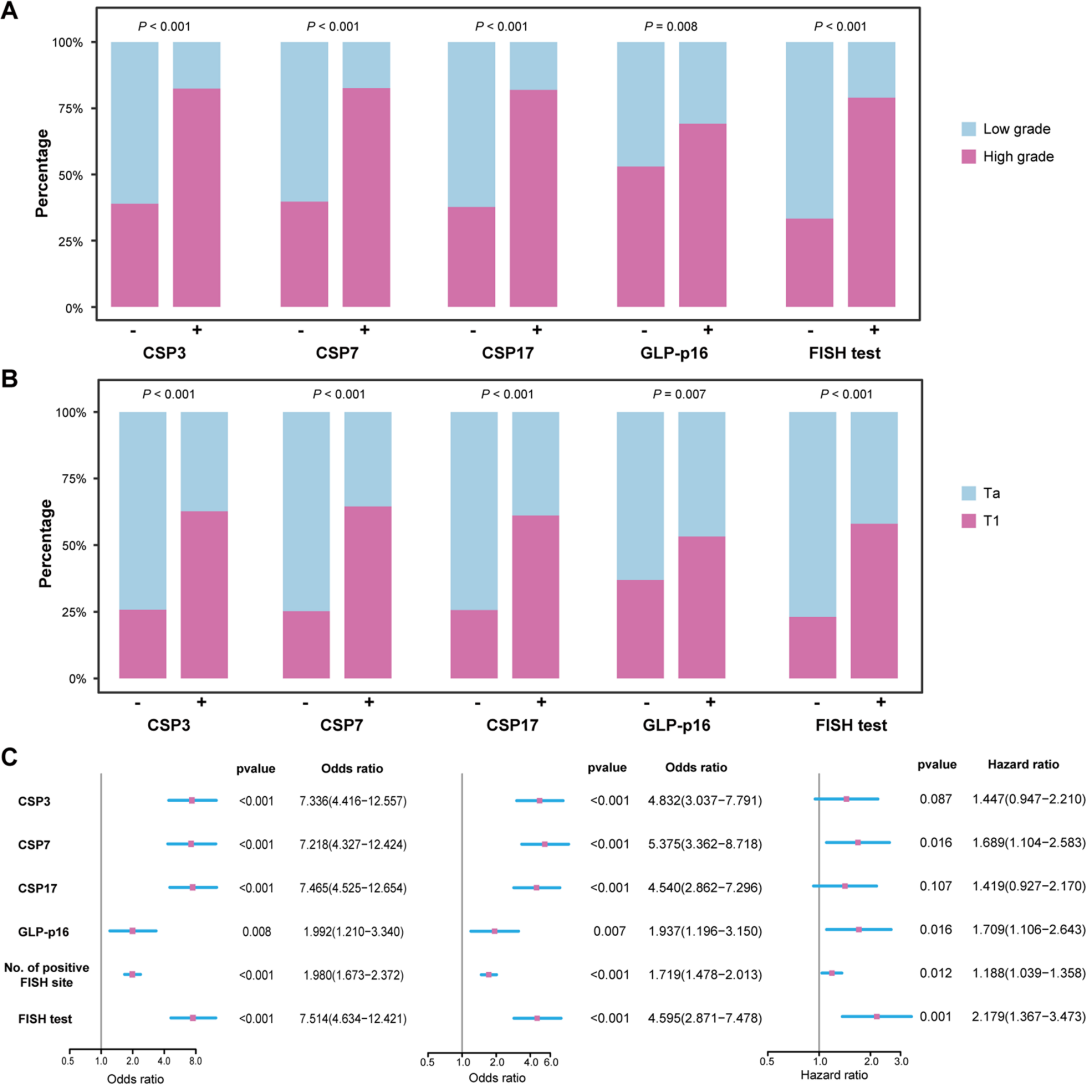
The correlations between the FISH assay results and tumor grade, stage as well as RFS. **A** Bar plots showing the correlations between the results of the FISH assay and tumor grade in the whole study cohort. **B** Bar plots showing the correlations between the results of FISH assay and tumor stage in the whole study cohort. **C** Forest plots showing the correlations between the results of FISH assay and tumor grade (left panel), stage (middle panel) as well as recurrence (right panel) in the whole study cohort

Our findings revealed that chromosome 3, 7, and 17 aneuploidies, p16 locus loss and the FISH test result had a significant correlation with tumor grade and stage in the whole study cohort (Chi-square tests, all *P* < 0.01, Fig. 2A and B). Furthermore, based on the logistic regression analyses, patients who had aneuploidy by chromosomes 3, 7, and 17, or p16 locus loss, or positive FISH test results, or a higher number of the positive FISH sites were more likely to suffer higher tumor grade and stage (Fig. 2C, left and middle panels). In addition, chromosome 7 aneuploidies, p16 locus loss, the number of the positive FISH sites, and the FISH test result were significantly associated with tumor recurrence (Fig. 2C, right panel). The analysis results in the SYSMH cohort and SYSUTH cohort are also presented in Additional file 1: Figs. S1 and S2, respectively. The Kaplan-Meier survival curves showed that patients with chromosome 7 aneuploidies (*P* = 0.014), p16 locus loss (*P* = 0.015), a higher number of the positive FISH sites (*P* < 0.001) or positive FISH test result (*P* < 0.001) had a shorter RFS in the whole study cohort. (Additional file 1 Fig. S3).

### Nomogram construction and performance assessment

**Fig. 3:**
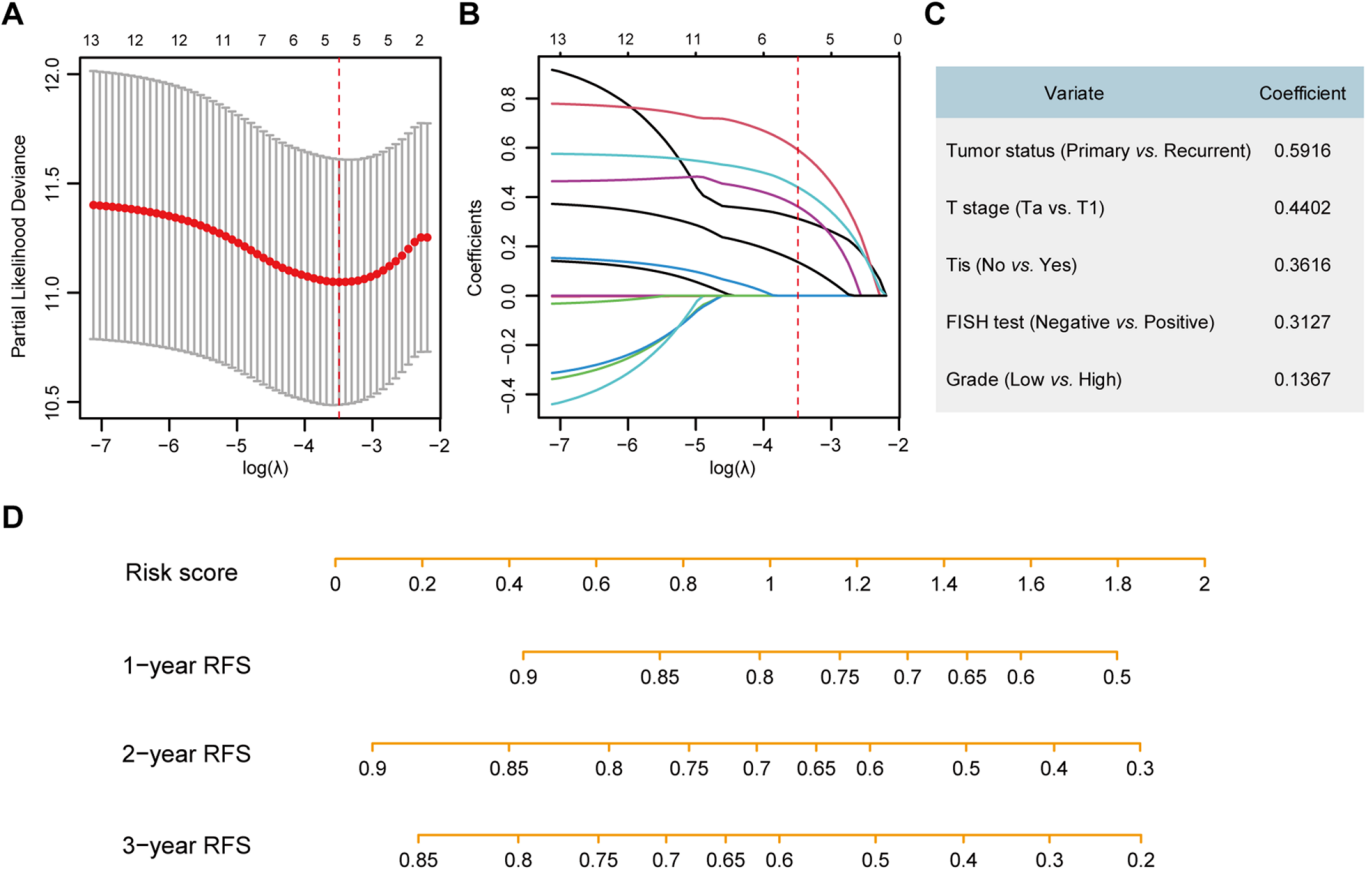
Construction of the FISH-clinical nomogram. **A** Tuning parameter selection (λ) with 10-fold cross-validation in the LASSO Cox model. The dotted vertical line is drawn at the optimal λ value by minimum criteria, which is 0.030 with log (λ) = − 3.493. **B** LASSO Cox coefficient profiles of the candidate variates. **C** Five selected predictors and their corresponding coefficients. **D** The FISH-clinical nomogram constructed based on the regression model is used to predict the 1, 2, and 3 years RFS rate for NMIBC patients. The risk score can be calculated according to the regression formula

**Fig. 4:**
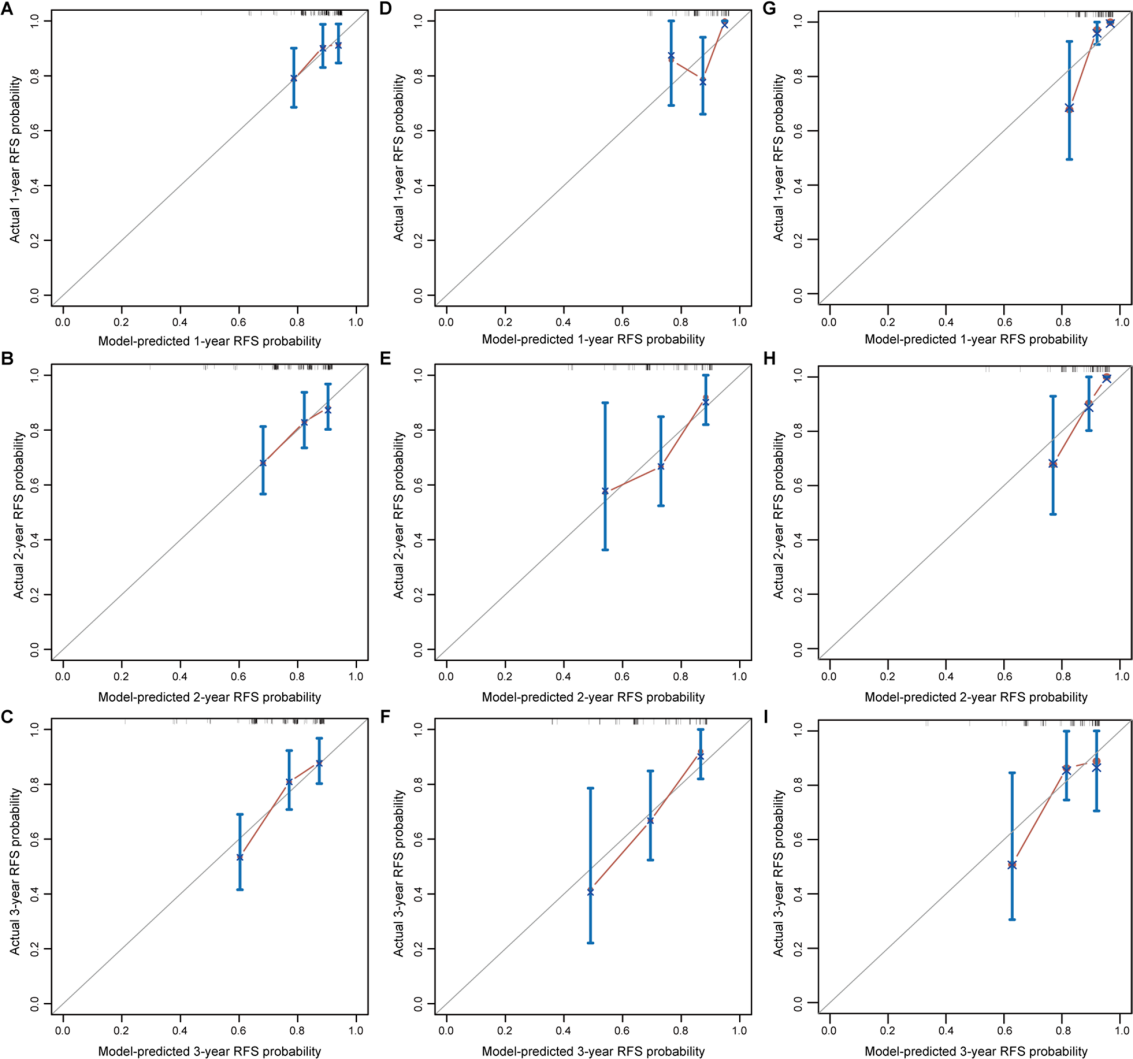
Calibration curves of the FISH-clinical nomogram. **A**–**C** Calibration curves of the FISH-clinical nomogram for 1, 2, and 3 year RFS prediction in the training set. **D**–**F** Calibration curves of the FISH-clinical nomogram for 1, 2, and 3 year RFS prediction in the validation set I. **G**–**I** Calibration curves of the FISH-clinical nomogram for 1, 2, and 3 year RFS prediction in the validation set II

By using the LASSO Cox regression algorithm, five recurrence-associated predictors including the FISH test result, T stage, associated CIS, tumor grade, and tumor status were identified (Fig. 3A and B), and their corresponding coefficients are shown in Fig. 3C. A FISH-clinical nomogram was developed based on the model, providing a user-friendly tool (Fig. 3D). In the training set, the FISH-clinical nomogram yielded a C-index of 0.683 (95%CI, 0.611–0.756), indicating favorable discrimination of the model. The calibration curves for the 1, 2, and 3 year RFS showed favorable agreement between the model-predicted RFS probability and actual RFS probability, indicating good calibration of the FISH-clinical nomogram in the training set (Fig. 4A–C).

### Validation of the nomogram

The good discrimination of the FISH-clinical nomogram was confirmed in the validation set I and the validation set II with C-indexes of 0.665 (95%CI, 0.565–0.765) and 0.778 (95%CI, 0.665–0.891), respectively. As shown in Fig. 4D–I, good calibration of the nomogram was also observed in two validation sets.

### Clinical usefulness of the nomogram

The DCA revealed that using the nomogram to predict RFS can provide greater net benefit than the “treat all” or “treat none” strategies in a wide range of threshold probabilities, suggesting the clinical usefulness of our proposed model (Additional file 1: Fig. S4). In addition, as shown in Additional file 1: Table S2, when compared with the EORTC and CUETO models, the FISH-clinical model demonstrated a smaller Akaike information criterion (AIC) and larger C-index, indicating that the performance was improved by adding the FISH assay to the model.

### Categorization of patients into high or low risk groups

**Fig. 5:**
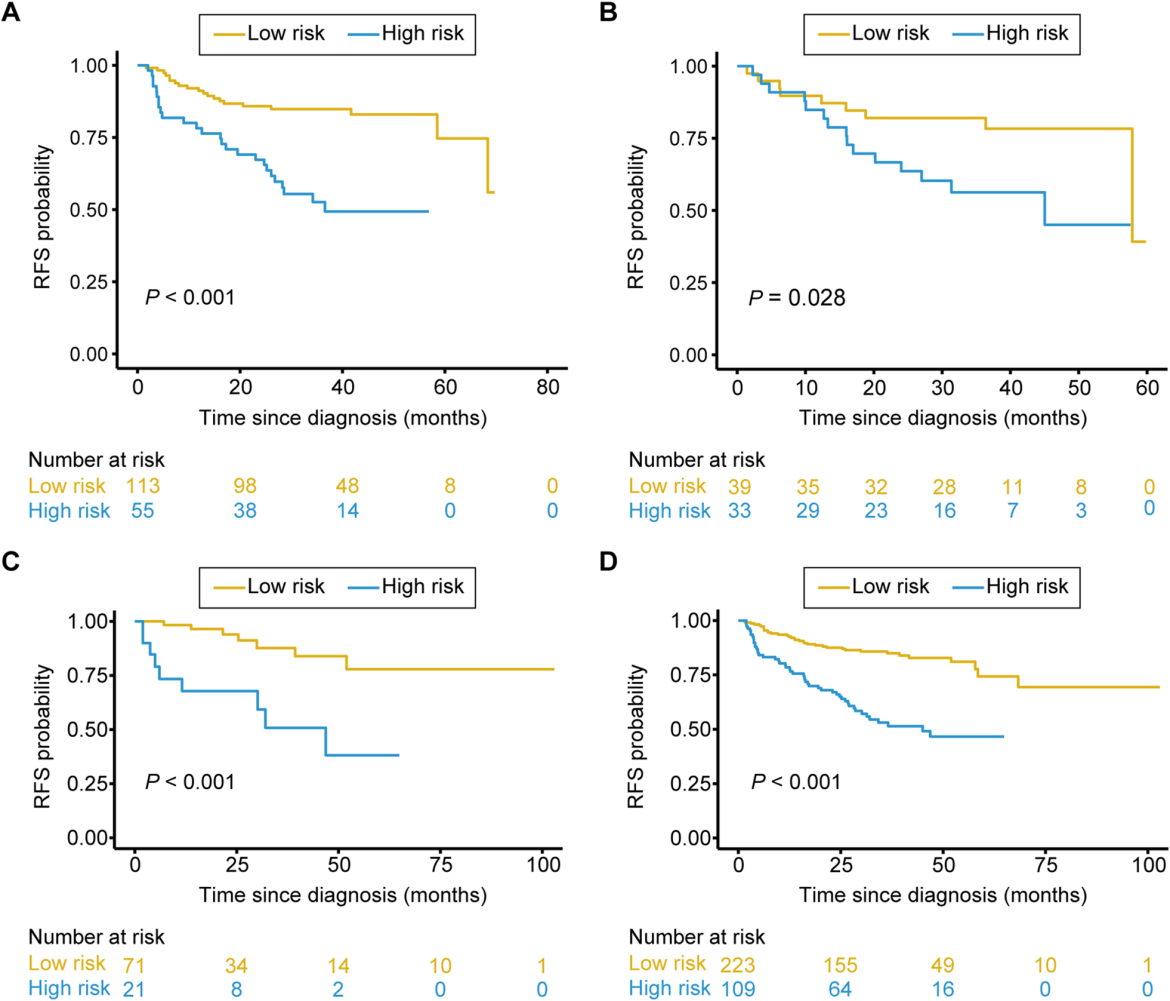
Kaplan-Meier survival curves of RFS between the low-risk and high-risk groups. The Kaplan-Meier survival curves showed that patients in the high-risk group exhibited worse RFS in the training set (**A**), validation set I (**B**), validation set II (**C**) and all enrolled patients (**D**)

The optimal risk score cutoff value generated by the X-tile plots was 0.82 (Additional file 1: Fig. S5). Based on the cutoff, all patients were divided into high risk and low risk groups. There was significant discrimination between the RFS of the high risk and low risk patients in the training set (Fig. 5A), which was confirmed in the validation set I (Fig. 5B), the validation set II (Fig. 5C) and the whole study cohort (Fig. 5D). Furthermore, the risk score was also related to the RFS in the stratified analyses (Additional file 1: Fig. S6). Therefore, the present FISH-clinical nomogram can successfully identify patients with a high risk of recurrence from those with low risk.

## Discussion

Our study uncovered that NMIBC patients with positive FISH test results might have higher tumor stage, grade, and worse RFS. Then, the FISH-clinical nomogram incorporating the FISH test result, T stage, associated CIS, tumor grade, and tumor status was further constructed for predicting the recurrence of NMIBC patients after operation. The nomogram had favorable prediction capability, which was superior to the guideline-recommended scoring models.

NMIBC is characterized by a high rate of recurrence and progression, despite proper treatment. Patients should follow a long-term follow-up screening, including regular cystoscopy starting at 3 months after tumor resection. NMIBC patients who progress to muscle invasive bladder cancer (MIBC) will have a higher probability of progression to metastatic disease, and once this occurs, their prognosis is worse [2]. Thus, precise stratification of recurrence risk after surgery is necessary for personalized treatment and follow-up strategies, which may improve patient outcomes. The current EAU guidelines recommended the EORTC and CUETO scoring models for risk stratification in patients with NMIBC, even though their performance is generally moderate, with C indexes of 0.66 and 0.636 for RFS prediction according to their original studies, respectively [6, 7]. Jobczyk et al. conducted a study to validate the reliability of these scoring models by using an independent cohort. As a result, the C indexes were only 0.64 and 0.53, respectively [8]. Therefore, it is crucial to search for new predictors to improve the RFS prediction in patients with NIMBC.

The conventional urine cytology test and the FISH assay are noninvasive methods for assisting in the diagnosis of bladder cancer in clinical practices [11, 22]. The urine cytology test has favorable specificity but poor sensitivity in the early stage of bladder cancer, and the FISH assay has been shown to have a higher sensitivity than cytology [23–25]. Thus, the FISH test can serve as an important auxiliary tool for detecting bladder tumors in patients with equivocal or negative cystoscopy and atypical cytology [26]. In our study, the NMIBC diagnostic sensitivities of the FISH test are 52.9% (127/240) and 53.3% (49/92) in the SYSMH cohort and SYSUTH cohort, respectively, which were similar to those of previous studies [27, 28]. Although the FISH test is a promising technique for bladder cancer detection, the false-negative rate is still relatively high in NMIBC patients. Therefore, the development of alternative or supplementary diagnostic methods is warranted to improve diagnostic accuracy, such as DNA methylation detection and nanotechnology [22, 29, 30].

In this study, we demonstrated that CSP3, 7, 17 aneuploidy and positive FISH test result were associated with higher tumor stage and grade in NMIBC, which is consistent with our previous study [16]. In that study, the positivity of CSP3, 7, and 17 in the FISH assay was found to be associated with muscular invasion in bladder cancer [16]. In addition, there have been other studies indicating that CSP7 and CSP17 aneuploidy were related to tumor stage in bladder cancer [31, 32]. Oncogenes on these chromosomes gain extra copy number may promote tumor proliferation, such as *EGFR* on chromosome 7 and *ERBB2* on chromosome 17 [31–34]. This may be one of the possible mechanisms of our findings. Furthermore, our study revealed that p16 locus loss was also associated with tumor stage and grade, which is in accordance with the previous study [35]. However, the opposite result was obtained in another study conducted by Berggren et al [36]. We hypothesize that these contradictory results might be related to the ethnicity of study subjects, which needs further investigation.

As we all know, the FISH assay also plays a crucial role in the surveillance of patients with NMIBC [11]. When the cystoscopy is negative and cytology is dubious, patients with a positive FISH assay which was performed during the surveillance process suffer a higher risk of recurrence [37, 38]. Previously, Lotan Y et al. found that a positive postoperative FISH test was associated with a 3.3-fold increased risk of recurrence^14^. Another study also has indicated that patients with a positive FISH test result in 3 months following TURBT and induction BCG therapy have a higher risk of developing tumor recurrence [39]. However, there is no research investigating whether the status of the FISH sites in the preoperative FISH assay can predict the risk of tumor recurrence in NMIBC so far. In our study, we demonstrated that p16 locus loss and chromosome 7 aneuploidies were associated with RFS in NMIBC patients. Indeed, previous studies have found that the loss of p16 detected from tumor tissue by immunohistochemistry and chromosome 9 monosomy detected from bladder irrigation specimens via FISH analysis were associated with tumor recurrence in bladder cancer [40, 41]. These indirectly proved the reliability of our findings. p16 is also named cyclin dependent kinase inhibitor 2 A (*CDKN2A*). Its loss might lead to cell proliferation, and the *CDKN2A* inactivation might be an early event in bladder carcinogenesis, indicating the loss of p16 would tend to recurrence [36, 40, 42]. Recently, *CDKN2A* as a negative regulator of cuproptosis, has been found to be associated with tumor prognosis and identified as a potential chemotherapy response predictor [43]. Whether there are other genes loss with chromosome 9 lead to this phenomenon deserves further investigation. Our findings provide novel insight into the bioinformatics analysis and biomedical basic research on bladder cancer, and are conducive to the discovery of new targets of anti-tumor therapy. Beyond the context of bladder cancer, our work may extend to other cancer types where chromosomal aberrations play a pivotal role. The principles laid out in our study could provide a framework for researchers exploring the relationship between chromosomal abnormalities and other malignancies, enhancing the broader applicability of our methodology.

In addition, we found that the risk of recurrence was more than doubled with a positive preoperative FISH test (HR = 2.179, *P* = 0.001, Fig. 2C), which is a stronger predictor of RFS in NMIBC than p16 locus loss and chromosome 7 aneuploidies. This result is inconsistent with the previous study by Chunjin K et al [27]. This might be related to the limitations of their study, including the small sample size (n = 69), relatively short median follow-up time (19.0 months), and enrolling seven MIBC patients in the study. On this basis, we further developed the FISH-clinical nomogram incorporating the FISH test result and other clinicopathological factors for RFS prediction in NMIBC after tumor resection, which successfully improved the prediction accuracy and was validated in an independent validation set. Moreover, the proposed nomogram significantly outperformed the guideline-recommended EORTC and CUETO scoring models in our study. Therefore, our model is expected to be an alternative to the existing methods. Certainly, the model still requires additional validation to verify its strength.

To our knowledge, our study is the first to elucidate the association of the status of the FISH sites in the preoperative FISH test with tumor stage, grade, and RFS in NMIBC patients. Moreover, we further innovatively introduced the FISH test result into RFS clinical prediction model, which improves the prediction capability. Notably, the FISH assay is a routine non-invasive medical test to assist in the diagnosis of bladder cancer in clinical practice, which can also be easily accessed. Therefore, our proposed FISH-clinical nomogram can serve as a novel tool for predicting the recurrence of NMIBC patients.

Despite these strengths, this study still contains several limitations. The limitations of our study include the retrospective study design. Although the FISH-clinical nomogram has been internally and externally validated, further external validation in larger cohorts and even prospective validation are still warranted to confirm the robustness of the model. Other promising techniques, such as radiomics, pathomics, and artificial intelligence, perhaps can also be combined to help improve the performance of the FISH-clinical nomogram [44–46]. Moreover, we revealed that the FISH assay results were significantly associated with tumor stage, grade, and recurrence in patients with NMIBC, but the underlying mechanism was not investigated in this study, which requires further investigation. In addition, copy number alterations could also be further explored to provide a more comprehensive genetic landscape of bladder cancer.

## Conclusions

In conclusion, our study demonstrated that the FISH assay results were associated with tumor stage, tumor grade, and RFS in NMIBC patients. In addition, the proposed FISH-clinical nomogram can be used to predict RFS in patients with NMIBC after surgery. This may optimize disease management and facilitate precision medicine in NMIBC patients.

### Supplementary Information

**Additional file 1: Table S1.** R packages used in our study. **Table S2.** Comparison of performance between different models. **Figure S1.** The correlations between the FISH assay results and tumor grade, stage as well as RFS in the SYSMH cohort. **Figure S2.** The correlations between the FISH assay results and tumor grade, stage as well as RFS in the SYSUTH cohort. **Figure S3.** Kaplan-Meier survival curves categorized by the status of CSP7, GLP-p16, number of positive FISH sites, and FISH test results in the whole study cohort. **Figure S4.** Decision curve analysis of the FISH-clinical nomogram for the 3-year RFS prediction of NMIBC patients. **Figure S5.** X-tile plots identifying the optimal risk score cutoff value based on RFS.** Figure S6.** Kaplan-Meier survival curves of RFS between the low-risk and high-risk groups in stratified analyses in the whole study cohort.

## Data Availability

The datasets used and/or analyzed during the current study are available from the corresponding author on reasonable request.

## Abbreviations

AIC: Akaike information criterion
CIS: Carcinoma in situ
CSP: Chromosome-specific centromeric probe
CUETO: The Club Urologico Espanol de Tratamiento Oncologico
DCA: Decision curve analysis
EORTC: The European Organisation for Research and Treatment of Cancer
FISH: Fluorescence in situ hybridization analysis
GLP: Gene locus-specific probe
LASSO: Least absolute shrinkage and selection operator
NMIBC: Non-muscle invasive bladder cancer
RFS: Recurrence-free survival
SYSMH: Sun Yat-Sen Memorial Hospital
SYSUTH: The Third Affiliated Hospital of Sun Yat-Sen University
TURBT: Transurethral resection of bladder tumor
