# Supplementary material for: Preoperative fluorescence in situ hybridization analysis as a predictor of tumor recurrence in patients with non-muscle invasive bladder cancer: a bi-institutional study
Source: J Transl Med. 2023 Oct 2;21:685. doi: 10.1186/s12967-023-04528-2 (PMC10546664; doi:10.1186/s12967-023-04528-2)

Supplementary Material

SUPPLEMENTARY METHODS 2

SUPPLEMENTARY TABLES 8

Table S1. R packages used in our study. 8

Table S2. Comparison of performance between different models. 9

SUPPLEMENTARY FIGURES 10

Figure S1. The correlations between the FISH assay results and tumor grade, stage as well as RFS in the SYSMH cohort. 10

Figure S2. The correlations between the FISH assay results and tumor grade, stage as well as RFS in the SYSUTH cohort. 11

Figure S3. Kaplan-Meier survival curves categorized by the status of CSP7, GLP-p16, number of positive FISH sites, and FISH test results in the whole study cohort. 12

Figure S4. Decision curve analysis of the FISH-clinical nomogram for the 3-year RFS prediction of NMIBC patients. 13

Figure S5. X-tile plots identifying the optimal risk score cutoff value based on RFS. 14

Figure S6. Kaplan-Meier survival curves of RFS between the low-risk and high-risk groups in stratified analyses in the whole study cohort. 15

SUPPLEMENTARY METHODS

1. Preparation of relevant reagents

(1) Buffer storage solution (20x Saline Sodium Citrate buffer [SSC], pH 5.3): add together 44 g sodium citrate, 88 g sodium chloride, and 400 mL deionized water. Mix thoroughly. At room temperature, adjust pH to 5.3 with 12M HCl, and lead to a final volume of 500 mL with deionized water.

(2) Buffer solution (2x SSC, pH 7.0 ± 0.2): add together 100 mL 20x SSC (pH 5.3), 800 mL deionized water. Mix thoroughly. At room temperature, adjust pH to 7.0 ± 0.2 with 10M NaOH, and lead to a final volume of 1 L with deionized water.

(3) 70% and 85% Ethanol solution: 700 mL and 850 mL 100% ethanol, and lead to a final volume of 1 L with deionized water, respectively.

(4) Denaturing Solution (70% formamide/2x SSC pH 7.0 ~ 8.0): add together 5 mL 20x SSC (pH 5.3) and 35 mL formamide. Mix thoroughly. At room temperature, adjust pH to 7.0 ~ 8.0, and lead to a final volume of 50 mL with deionized water.

(5) KCl hypotonic solution: add together 2.795 g KCl and 300 mL deionized water. Mix thoroughly. At room temperature, lead to a final volume of 500 mL with deionized water.

(6) 1M HCl: add together 8.2 mL concentrated HCl and 80 mL deionized water. Mix thoroughly. At room temperature, lead to a final volume of 100 mL with deionized water.

(7) Formamide washing solution (50% formamide/2x SSC): add together 75mL formamide and 15 mL 20x SSC (pH 5.3). Mix thoroughly. At room temperature, adjust pH to 7.0 ~ 8.0, and lead to a final volume of 150 mL with deionized water.

(8) 0.01M HCl: 0.4 mL 1M HCl. At room temperature, lead to a final volume of 40 mL with deionized water.

(9) Pepsin (0.02 mg/mL): warm 40 mL 0.01M HCl up to 37 °C and add 0.8 mg powder pepsin. Mix thoroughly.

(10) 0.4x SSC/0.3% NP-40: add together 20 mL 20x SSC (pH 5.3), 3 mL NP-40, and 950 mL deionized water. Mix thoroughly. At room temperature, adjust pH to 7.0 ~ 7.5 with 10M NaOH and lead to 1 L with deionized water.

(11) Fixative (3:1, methanol–acetic acid): add together 30 mL methanol and 10 mL glacial acetic acid. Mix thoroughly.

(12) 0. 1M HCl: 4 mL 1M HCl. At room temperature, lead to a final volume of 40 mL with deionized water.

(13) 2x SSC/0.1% NP-40: bring together 40 mL 20x SSC (pH 5.3), 0.4 mL NP-40 and 300 mL deionized water. Mix thoroughly. At room temperature adjust pH to 7.0 ± 0.2 with 10M NaOH and lead to 400 mL with deionized water.

2. Experimental procedure

2.1 Slide preparation procedure

2.1.1 Specimen Collection:

About 200 mL of voided urine specimens (morning urine is preferred) are collected for FISH detection. If urine is not shipped immediately after collection, refrigerate immediately and ship within 24 h. Otherwise, the sample should be discarded.

2.1.2 Specimen Processing:

(1) Centrifuge urine in a 50 mL centrifuge tube at 1500~2000 rpm for 10 min. Centrifuge repeatedly, remove supernatant and collect cells. Note: Collagenase B digestion can be carried out according to the situation after the collection of cells (e.g., specimen protein, blood cells, mucus, impurities, etc.).

(2) Hypotonic: Add 5mL 0.075M KCI solution preheated to 37°C, hypotonic at 37°C for 20 min, during which the suspended cells are gently blown 2-3 times with a glass blow pipe. Prefixation: Slowly add 2ml fixative (3:1, methanol–acetic acid), then gently blow and mix, and finally centrifuge at 1000 rpm for 10 min. Fixation: Remove supernatant, add 5ml fixative, Mix

thoroughly. Let stand for 10 min, blow once during the period, and centrifuge at 1000 rpm for 10 min.

(3) Preparation and preservation of cell suspension: centrifuge, remove supernatant, and add appropriate amount of fixative according to the number of cells. Mix thoroughly. Apply cell suspension on the slide. Air-dry the samples: Bake at room temperature overnight or 56°C for at least 2 hours. Dry and store at room temperature.

(4) Immerse slides in 2x SSC (pH 7.0) solution at room temperature for 5min. Digestion: immerse slides in pepsin at 37°C for 10 min, which can be adjusted according to the actual situation. Then immerse slides in 2x SSC (pH 7.0) solution at room temperature for 5min.

(5) The slides were dehydrated in 70% ethanol solution, 85% ethanol solution, and 100% ethanol solution for 3min respectively, and then dried naturally. Probe preparation. Centrifuge for 1 to 3 seconds, vortex mixing, and centrifuge again briefly.

(6) Denaturation and hybridization. The 10 uL probe mixture was first dripped onto the slide hybridization area, then the coverslip was immediately covered, and finally, the edge is sealed with rubber cement. Codenaturation in hybridizer. Denaturation condition: 75 °C, 5 min; hybridization condition: 42°C, 16 h. Hybridization buffer 7.0 uL, deionized water 1.0 uL, probe 2.0 uL.

2.2 Post-hybridization washes and Counterstaining

(1) Fill a Coplin jar with 0.4x SSC/0.3% NP-40 and place in a 67°C constant temperature water bath for at least 30 min prior to washing. The temperature of the solution should be 67°C.

(2) After removing the coverslip, place slices in the 0.4x SSC/0.3% NP-40 solution. Full oscillation 1~3 sec, then washing 2 min. Note: The temperature and time of the wash solution should be adjusted according to the difference of specimen. 67 °C is the recommended temperature.

(3) At room temperature, place slices in the 2x SSC/0.1% NP-40. Full oscillation 1~3 sec, then washing 30 sec.

(4) At room temperature, place slices in the 70% ethanol solution then full wash for 3 min.

(5) Remove the slides from the wash solution and place them vertically in a dark area on a paper towel to dry completely.

(6) Apply 15 μL of DAPI counter stain on the target of the slide, then place a coverslip immediately on the slide. Store the slides in the dark for 10~20 min prior to signal evaluation.

2.3 Threshold establishment

(1) Urine specimens were collected from 10 people who were excluded from urinary tract tumors.

(2) The FISH experiment was performed according to the instructions manual, and 100 cells with clear signals were observed with each probe combination. The threshold was established by the percentage of cells with different types of abnormal conditions was counted.

(3) Threshold = mean(M) ± 3 * standard deviation (SD)

(4) The thresholds of the following five indicators are established, which are denoted as +3, +7, +17, -P16(-1), -P16(-2)：

- +3 indicator abnormality refers to the presence of three or more green signal points in a single cell, indicating polysomy of chromosome 3.
- +7 indicator abnormality refers to the presence of three or more red signal points in a single cell, indicating polysomy of chromosome 7.
- +17 indicator abnormality refers to the presence of three or more green signal points in a single cell, indicating polysomy of chromosome 17.
- -P16(-1) indicator abnormality refers to the presence of a red signal point in a single cell, indicating the loss of P16 heterozygosity
- -P16(-2) indicator abnormality refers to the absence of red signal points in a single cell, indicating the absence of the homozygous P16 gene, which is a complex abnormality

2.4 Indicator positive judgment

Each probe combination counts 100 cells with clear signals. First judges the indicators, and then judges the FISH results according to the principles.

(1) Negative indicator: The detection indicator value is less than the threshold.

(2) Positive indicator: The detection indicator value (+3, +7, +17) ≥ 10%; [-P16(-1)] ≥ 15%

(3) Gray area: When the value of the detection indicator is greater than or equal to the threshold but less than 10% (+3, +7, +17) or 15% [-P16(-1), -P16(-2)], morphological methods were considered for evaluation.

2.5 FISH positive judgment

(1) Two or more indicators are abnormal simultaneously.

(2) [-P16(-2)] ≥ 15%.

(3) Only one indicator is abnormal, refer to the morphological interpretation method.

2.6 Morphological interpretation method

Precondition: The experimenter has a certain morphological knowledge, the number of cells available to count is less than 100, or the threshold method results in the gray area.

To analyze the shed cells with abnormal morphology, at least 25 cells should be analyzed in each probe combination. If the results are still uncertain, the number of analyzed cells can be increased without capping.

2.6.1 Indicator positive judgment

The thresholds of the following four indicators are established, which are denoted as +3, +7, +17, -P16(-1):

- +3: ≥ 4 cells of chromosome 3 polysomy (three or more green signal points in a single cell)
- +7: ≥ 4 cells of chromosome 7 polysomy (three or more red signal points in a single cell)
- +17: ≥ 4 cells of chromosome 17 polysomy (three or more green signal points in a single cell)
- -P16(-1): ≥12 cells heterozygosity deletion of P16 gene (one red signal point in a single cell)

2.6.2 FISH positive judgment

(1) Two or more indicators are abnormal simultaneously.

(2) An indicator of complex abnormality: there are at least two or more abnormal signal patterns in the same chromosome (the number of signal points, such as 3 signal points, 5 signal points are present), and the number of cells in each abnormal signal pattern is ≥ 4.

(3) ≥12 cells have -P16(-2) (no red signal points in a single cell).

SUPPLEMENTARY TABLES

Table S1. R packages used in our study.

Statistical analysis	R package
Forest plot	forestplot
Cox regression analysis, log-rank test	survival, survminer
LASSO Cox regression	glmnet
nomogram construction, C-index calculation, and calibration plot	rms
C-index comparison	Hmisc
Decision curve analysis	dca.R

Table S2. Comparison of performance between different models.

Models	Whole study cohort			SYSMH cohort			SYSUTH cohort		
	**AIC**	**C-index (95% CI)**	***P****	**AIC**	**C-index (95% CI)**	***P****	**AIC**	**C-index (95% CI)**	***P****
**FISH-clinical model**	894.15	0.698 (0.646 - 0.750)		691.75	0.680 (0.622 - 0.738)		117.23	0.778 (0.665- 0.891)	
**EORTC model**	912.18	0.638 (0.585 – 0.690)	0.002	705.58	0.617 (0.557 - 0.677)	0.011	121.54	0.689 (0.567 - 0.811)	0.010
**CUETO model**	906.02	0.638 (0.577- 0.699)	0.002	699.24	0.634 (0.568 - 0.700)	0.041	120.88	0.686 (0.549 – 0.823)	0.013

AIC: Akaike information criterion.

* *P* values were derived by comparing the FISH-clinical model with the EORTC and CUETO scoring models with respect to the C-index.

SUPPLEMENTARY FIGURES

Figure S1. The correlations between the FISH assay results and tumor grade, stage as well as RFS in the SYSMH cohort.

(A) Bar plots showing the correlations between the results of the FISH assay and tumor grade. (B) Bar plots showing the correlations between the results of FISH assay and tumor stage. (C) Forest plots showing the correlations between the results of FISH assay and tumor grade (left panel), stage (middle panel) as well as recurrence (right panel).

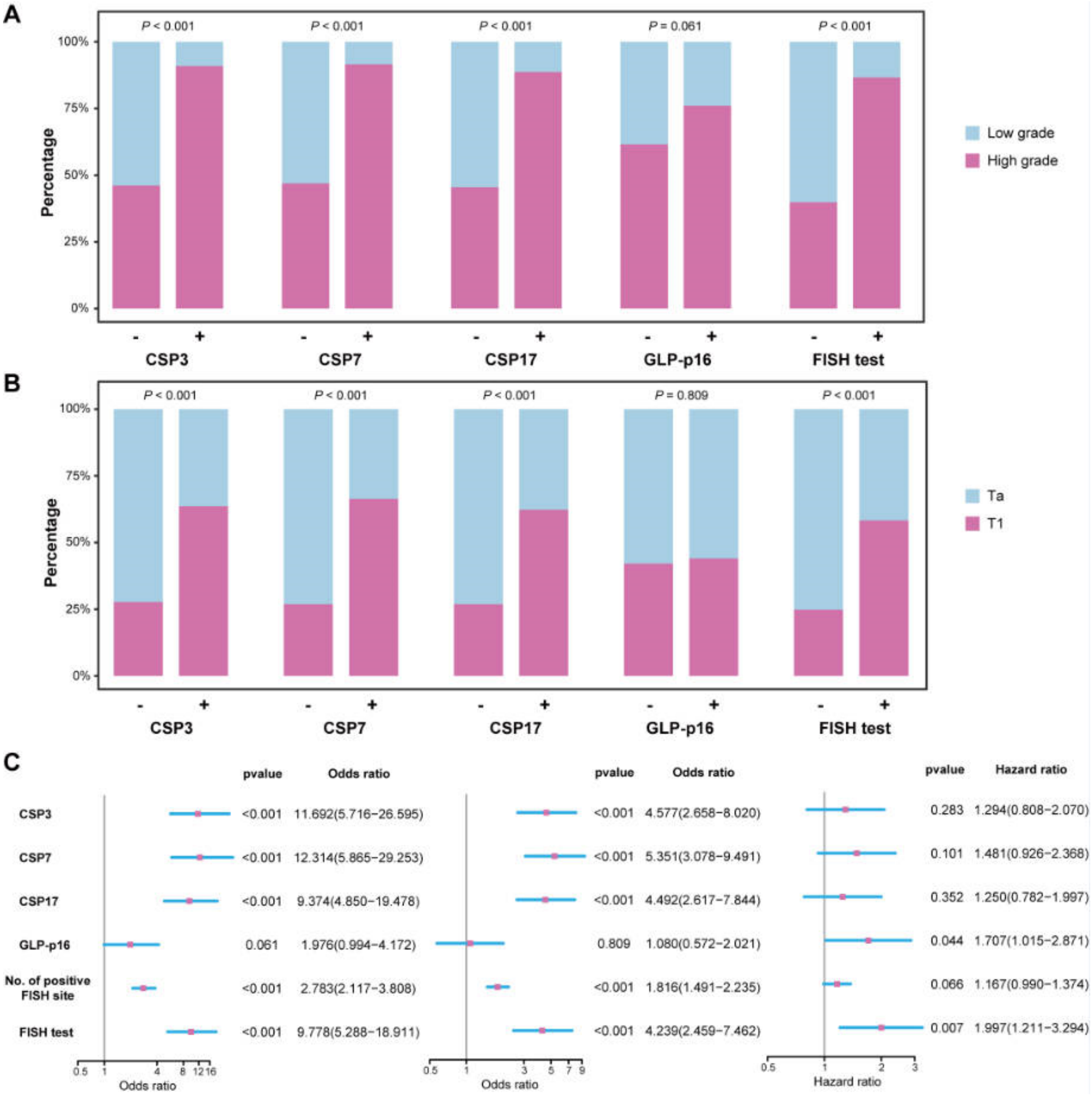

Figure S2. The correlations between the FISH assay results and tumor grade, stage as well as RFS in the SYSUTH cohort.

(A) Bar plots showing the correlations between the results of the FISH assay and tumor grade. (B) Bar plots showing the correlations between the results of FISH assay and tumor stage. (C) Forest plots showing the correlations between the results of FISH assay and tumor grade (left panel), stage (middle panel) as well as recurrence (right panel).

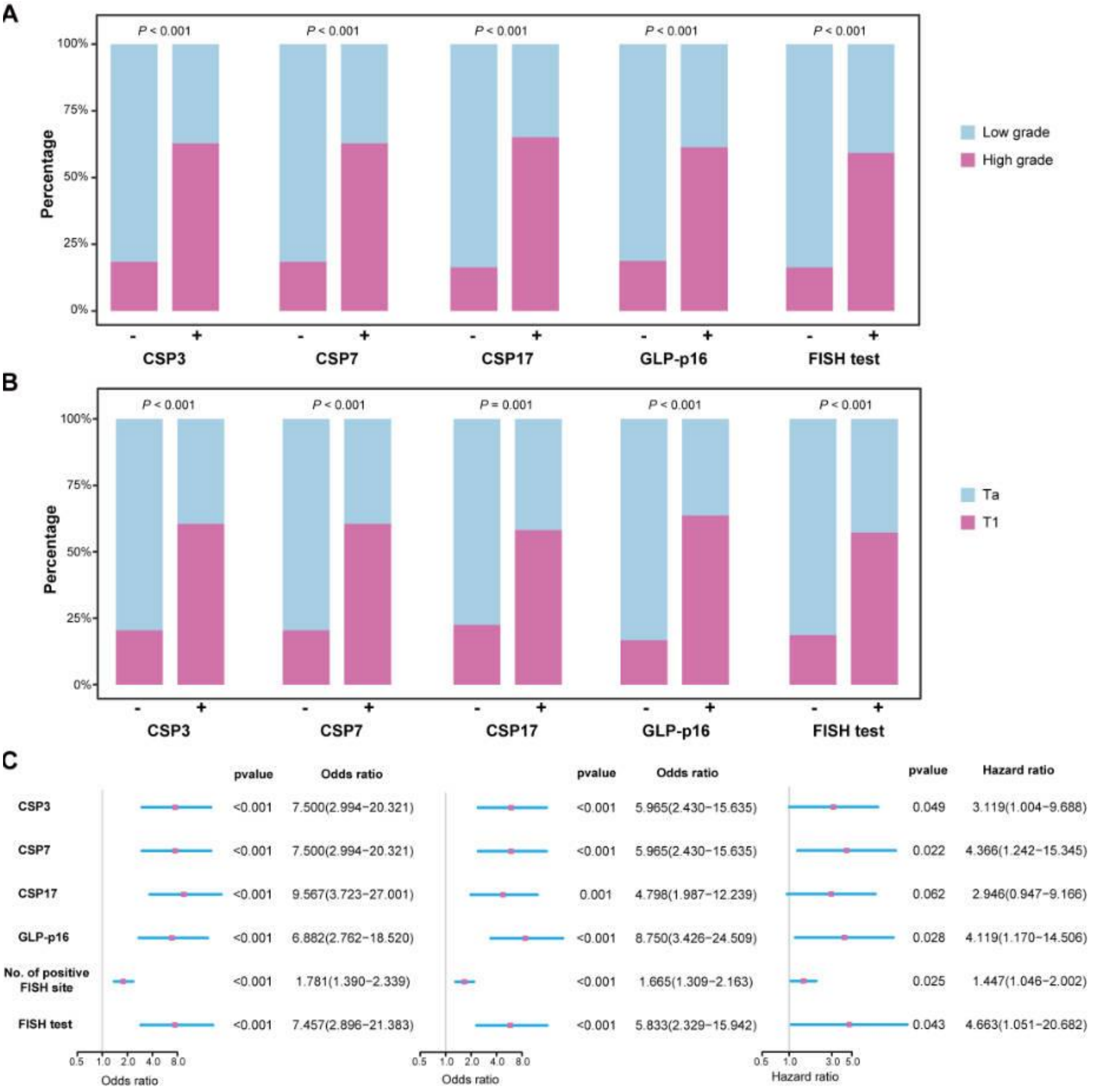

Figure S3. Kaplan-Meier survival curves categorized by the status of CSP7, GLP-p16, number of positive FISH sites, and FISH test results in the whole study cohort.

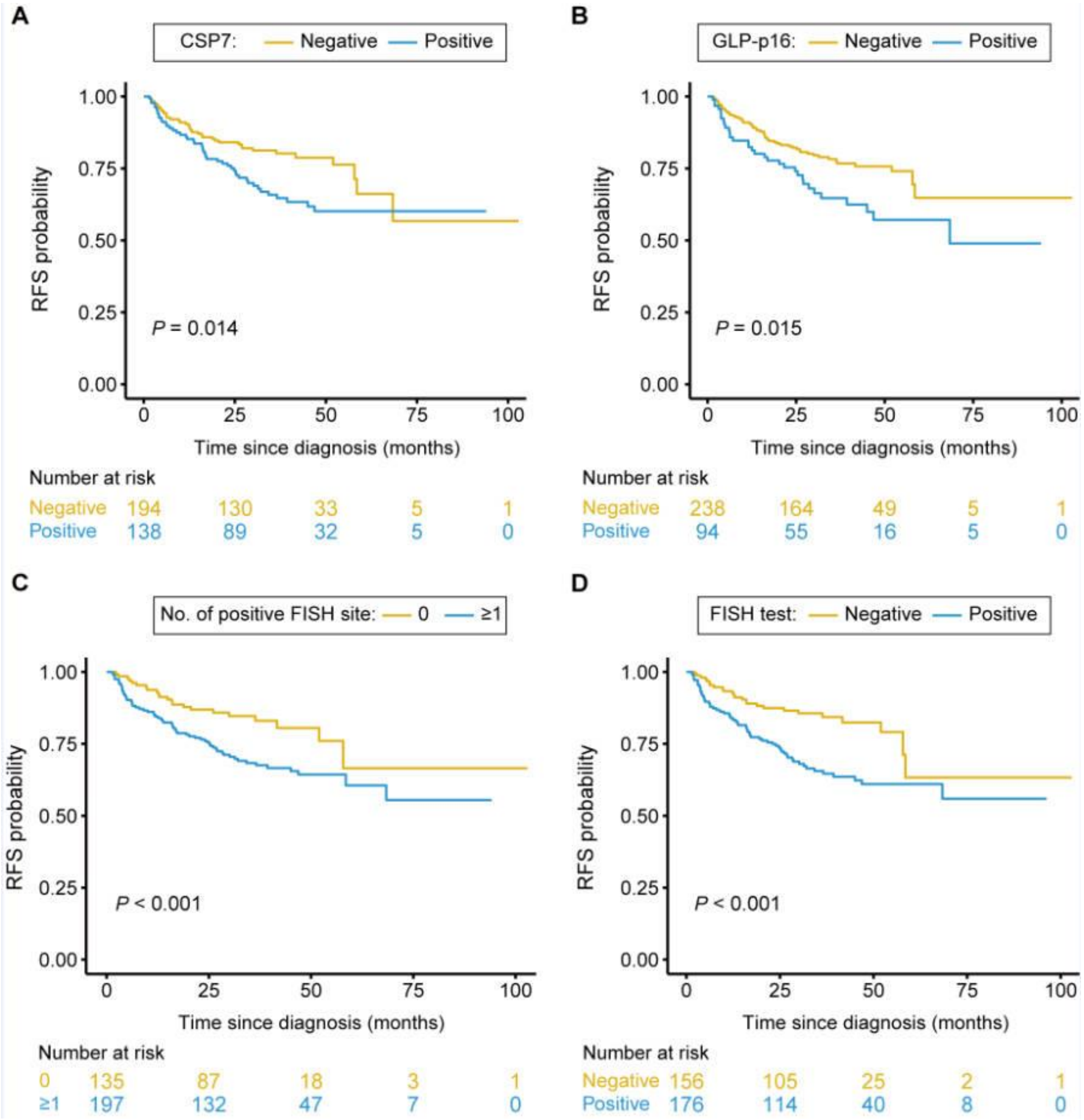

Figure S4. Decision curve analysis of the FISH-clinical nomogram for the 3-year RFS prediction of NMIBC patients.

(A) DCA in the validation set I. (B) DCA in the validation set II. The decision curve presents the net benefit versus the threshold probability. The red line refers to the nomogram. The gray line refers to the assumption that all patients had a recurrence, while the black line represents the assumption that no patients had a recurrence.

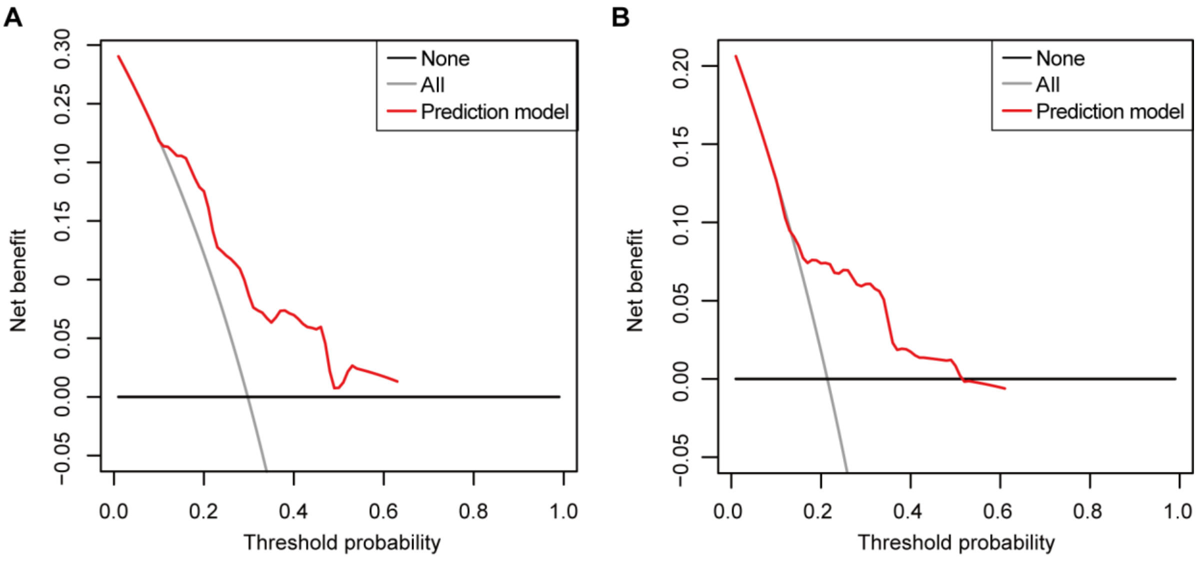

Figure S5. X-tile plots identifying the optimal risk score cutoff value based on RFS.

(A) X-tile plots for the training set. The coloration of the plot represents the strength of the association at each division, ranging from low (dark, black) to high (bright, red). (B) The distributions of the number of patients by risk score. (C) Kaplan-Meier plots categorized by the low-risk and high-risk groups according to the optimal risk score cutoff. X-tile plots identifying the optimal RFS risk score cutoff. The optimal RFS risk score cutoff was determined as 0.820 (χ^2 = 19.073, P <0.001).

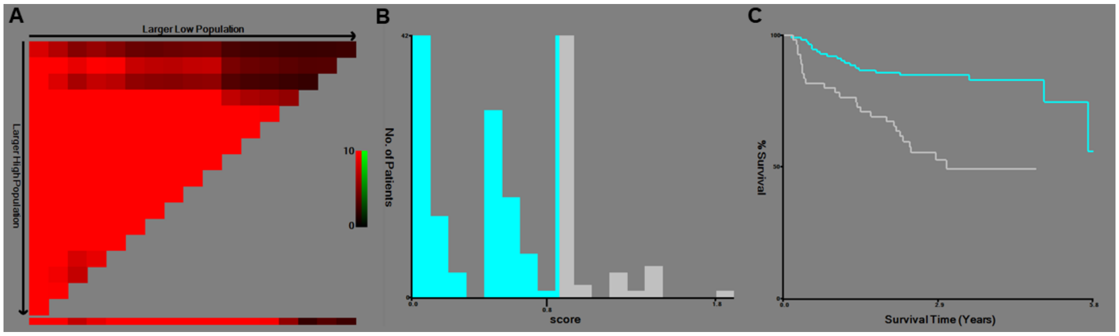

Figure S6. Kaplan-Meier survival curves of RFS between the low-risk and high-risk groups in stratified analyses in the whole study cohort.

Kaplan-Meier survival curves indicated that patients in the high-risk group have shorter RFS than patients in the low-risk group, which have the same consequence in various subgroups, including age (A, B) and sex (C, D).

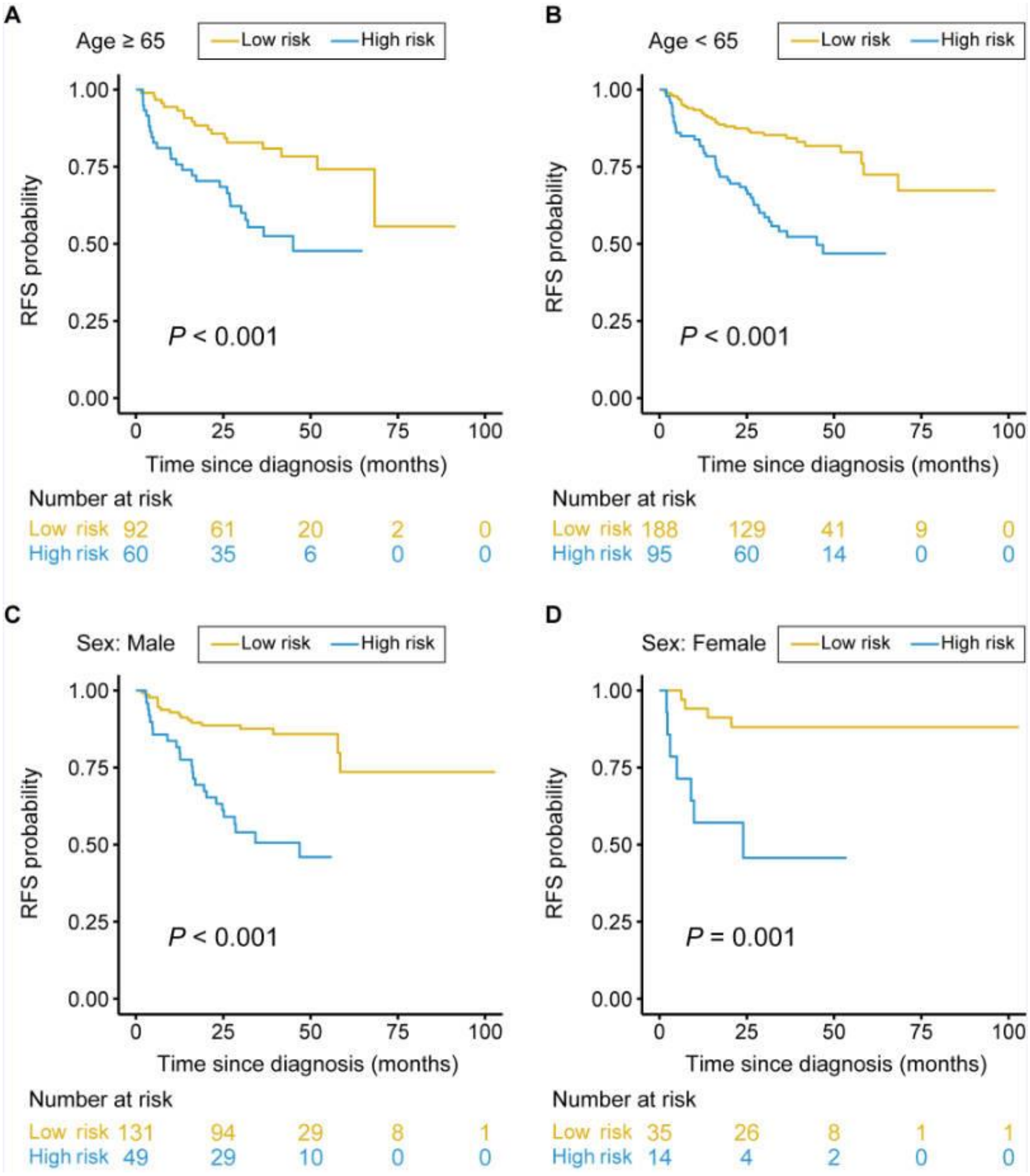

Supplement: Supplementary file 1 — Additional file 1: Table S1. R packages used in our study. Table S2. Comparison of performance between different models. Figure S1. The correlations between the FISH assay results and tumor grade, stage as well as RFS in the SYSMH cohort. Figure S2. The correlations between the FISH assay results and tumor grade, stage as well as RFS in the SYSUTH cohort. Figure S3. Kaplan-Meier survival curves categorized by the status of CSP7, GLP-p16, number of positive FISH sites, and FISH test results in the whole study cohort. Figure S4. Decision curve analysis of the FISH-clinical nomogram for the 3-year RFS prediction of NMIBC patients. Figure S5. X-tile plots identifying the optimal risk score cutoff value based on RFS. Figure S6. Kaplan-Meier survival curves of RFS between the low-risk and high-risk groups in stratified analyses in the whole study cohort. [file 12967_2023_4528_MOESM1_ESM.pdf]
